# Characterization of Invasive *Salmonella* Serogroup C1 Infections in Mali

**DOI:** 10.4269/ajtmh.17-0508

**Published:** 2017-12-26

**Authors:** Fabien J. Fuche, Sunil Sen, Jennifer A. Jones, Joseph Nkeze, Jasnehta Permala-Booth, Milagritos D. Tapia, Samba O. Sow, Boubou Tamboura, Aliou Touré, Uma Onwuchekwa, Mamadou Sylla, Karen L. Kotloff, Sharon M. Tennant

**Affiliations:** 1 Center for Vaccine Development and Institute for Global Health, University of Maryland School of Medicine, Baltimore, Maryland,; 2 Department of Medicine, University of Maryland School of Medicine, Baltimore, Maryland,; 3 Department of Pediatrics, University of Maryland School of Medicine, Baltimore, Maryland;; 4 Centre pour le Développement des Vaccins, Mali, Bamako, Mali

## Abstract

Nontyphoidal *Salmonella* (NTS) are the leading cause of foodborne infections worldwide and a major cause of bloodstream infections in infants and HIV-infected adults in sub-Saharan Africa (SSA). *Salmonella* Typhimurium (serogroup B) and *Salmonella* Enteritidis (serogroup D) are the most common serovars in this region. However, data describing rarer invasive NTS serovars, particularly those belonging to serogroups C1 and C2, circulating in SSA are lacking. We previously conducted systematic blood culture surveillance on pediatric patients in Bamako, Mali, from 2002 to 2014, and the results showed that serovars Typhimurium and Enteritidis accounted for 32% and 36% of isolates, respectively. Here, we present data on 27 *Salmonella* serogroup C1 strains that were isolated during this previous study. The strains were typed by serum agglutination and multilocus sequence typing (MLST). Sixteen strains were *Salmonella* Paratyphi C, four were *Salmonella* Colindale, and two were *Salmonella* Virchow. Interestingly, five strains were identified as the very rare *Salmonella* Brazzaville using a combination of serum agglutination and flagellin gene typing. Phenotypic characterization showed that *Salmonella* Brazzaville produced biofilm and exhibited catalase activity, which were not statistically different from the gastroenteritis-associated *Salmonella* Typhimurium sequence type (ST) 19. All tested *Salmonella* Paratyphi C strains were poor biofilm producers and showed significantly less catalase activity than *Salmonella* Typhimurium ST19. Overall, our study provides insight into the *Salmonella* serogroup C1 serovars that cause invasive disease in infants in Mali. In addition, we show that MLST and flagellin gene sequencing, in association with traditional serum agglutination, are invaluable tools to help identify rare *Salmonella* serovars.

## INTRODUCTION

Nontyphoidal *Salmonella* (NTS) are the leading cause of foodborne infections worldwide and a major cause of gastroenteritis and bloodstream infections. Of the 94 million cases and 155,000 deaths attributed to NTS every year worldwide, sub-Saharan Africa (SSA) bears the highest burden with 193–338 disability-adjusted life years per 100,000 individuals, compared with 50 and 67 for Europe and North America, respectively.^1^ In particular, invasive NTS (iNTS) disease, in which bacteria invade the bloodstream and cause life-threatening disseminated infections, is a leading cause of morbidity and mortality among infants and HIV-positive adults in SSA, with up to a 30% mortality rate.^2,3^

We recently emphasized the underestimated burden of NTS infections caused by *Salmonella enterica* serogroups C1 (O:6,7) and C2 (O:6,8).^4^ Although *Salmonella* serovars Typhimurium (serogroup B; O:4) and Enteritidis (serogroup D; O:9) have been extensively recognized as the major serovars responsible for iNTS infections in SSA, little is known about serovars from serogroup C1 or C2. Yet the prevalence of these two serogroups has been increasing worldwide (from 22.5% to 34.7% of NTS in the United States and from 5% to 8.6% in Europe, between 1995 and 2012), including in Africa where they now represent 19.5% of all reported NTS cases. Large studies that describe the *Salmonella* serovar distribution in Africa are needed to better understand the burden of iNTS disease.

We have been conducting systematic blood culture surveillance at l’Hôpital Gabriel Touré, the main teaching hospital in Bamako, Mali, since 2002.^5^ This work has provided valuable information about the burden of invasive bacterial pathogens in pediatric patients, such as *Haemophilus influenzae* type b and *Streptococcus pneumoniae*, as well as *S. enterica*.^6–9^ In the period of 2002–2014, 687 NTS isolates were obtained from blood samples of 667 children.^5^ *Salmonella* Enteritidis (36%), *Salmonella* Typhimurium (32%), *Salmonella* I 4,[5],12:i:-(6%), and *Salmonella* Dublin (13%) were the most common serovars. In addition, 27 serogroup C1 or C2 strains were isolated, but were not serotyped further. Here, we describe the identification of *Salmonella* serovars that previously agglutinated with O:6 antisera by biochemical and molecular analyses and also report phenotypic characteristics of these strains.

## MATERIALS AND METHODS

### Ethics statement.

The clinical research protocol and consent form were reviewed by the Ethics Committee of the Faculté de Médecine, de Pharmacie et d’Odonto-Stomatologie, University of Mali and by the Institutional Review Board of the University of Maryland, Baltimore. Consent was obtained from parents/guardians of every child and assent of the child was also required for enrollment of participants > 13 years of age.

### Bacterial strains.

Twenty-seven *Salmonella* strains that agglutinated with O:6 antisera were previously isolated from pediatric patients who presented with fever or clinical symptoms compatible with invasive bacterial disease at l’Hôpital Gabriel Touré in Bamako, Mali, between 2002 and 2014.^5^ Control strains for phenotypic assays included *S*. Typhimurium I77 (ST19) and D65 (ST313), *S*. Typhi Ty2, and *S.* Paratyphi A ATCC 9150 (American Type Culture Collection, Manassas, VA).^5,10^

### Bacterial growth conditions.

Unless otherwise specified, all bacterial strains were grown in Hy-Soy medium (10 g/L soytone [Teknova, Hollister, CA], 5 g/L hy-yest [Kerry Bio-Science, Norwich, NY], and 5 g/L sodium chloride [AmericanBio, Natick, MA]). When needed, agar (AmericanBio) was added at 15 g/L.

### Clinical microbiology.

NTS strains were agglutinated with O grouping and H typing antisera (Denka Seiken Co. Ltd, Tokyo, Japan and Difco, BD Diagnostics, Franklin Lakes, NJ), and serovars were identified according to the White–Kauffmann–Le Minor typing scheme.^11^

The ability to ferment dulcitol and mucate was assessed using dulcitol-containing phenol red broth (BD Diagnostics) and Remel mucate medium (Thermo Fisher Scientific, Lenexa, KS), and hydrogen sulfide production was determined by precipitation of sodium thiosulfate and ferric ammonium citrate after inoculation of triple sugar iron medium (EMD Chemicals, Billerica, MA). These biochemical characteristics then allowed us to discriminate between *Salmonella* serovars Paratyphi C (dulcitol-positive, H_2_S-positive, and mucate-negative), Choleraesuis sensu stricto (dulcitol-negative, H_2_S-negative, and mucate-negative), Choleraesuis var. Kunzendorf (dulcitol-negative, H_2_S-positive, and mucate-negative), and Choleraesuis var. Decatur (dulcitol-positive, H_2_S-positive, and mucate-positive).

Antimicrobial susceptibility was determined using the Kirby–Bauer disk diffusion method, as previously described.^5^ Antimicrobials tested were amikacin (30 μg), ampicillin (10 μg), aztreonam (30 μg), cefazolin (30 μg), cefepime (30 μg), streptomycin (10 μg), ciprofloxacin (5 μg), nalidixic acid (30 μg), tigecycline (15 μg), and sulfamethoxazole/trimethoprim (cotrimoxazole, 25 μg).

### Detection of *viaB* by polymerase chain reaction (PCR).

The presence of Vi antigen in *S.* Paratyphi C isolates was evaluated by PCR using 0.3 μM of each primer JN07-Vi_F (5′-GCACCGTTTAACCAACATCAAG-3′) and JN08-Vi R (5′-TGTACCTGCGCTGATGATCTG-3′), 5 U of Green Taq polymerase (GenScript, Piscataway, NJ), and 0.3 mM stabilized dNTP (GenScript) in a 50 μL reaction under the following cycling conditions: initial denaturation at 95°C for 3 minutes, 30 cycles of amplification (95°C for 30 seconds, 60°C for 45 seconds, and 70°C for 5 seconds), and a final elongation step at 95°C for 5 minutes.

### Multilocus Sequence Typing (MLST).

MLST was performed according to Achtman et al.^12^ Previously described primers were used to amplify seven loci (*aroC*, *dnaN*, *hemD*, *hisD*, *purE*, *sucA*, and *thrA*) from freshly extracted genomic DNA (GenElute Kit; Sigma-Aldrich, St. Louis, MO).^13^ Correct amplification was confirmed by agarose gel electrophoresis, and PCR fragments were gel-purified using a QIAquick gel extraction kit (Qiagen, Hilden, Germany). Sequencing data were obtained from Genewiz (South Plainfield, NJ) using described sequencing primers and applied to the MLST database search engine available at http://mlst.warwick.ac.uk/mlst/dbs/Senterica for allele and sequence type (ST) identification.

### Flagellin typing by PCR.

When needed, flagellar H-type was determined by amplifying the *fliC* gene using primers FLIC-F (5′-CAAGTCATTAATACAAACAGC-3′) and FLIC-R (5′-TTAACGCAGTAAAGAGAGGAC-3′) described by Weill et al.^14^ and the *fljB* gene using primers fljb-fw (5′-ATCAGGATCCTTCCAAAAGGAAA-3′) and fljb-rev (5′-TCAGCTAGCTGAATAAAACGAAA-3′) designed in this study. PCR assays were performed using 0.8 μM of each primer, 1 U of Vent DNA polymerase (New England Biolabs, Ipswich, MA), and 0.4 mM stabilized dNTP (GenScript) in a 50 μL reaction under the following cycling conditions: initial denaturation at 95°C for 2 minutes, 30 cycles of amplification (95°C for 30 seconds, 60°C for 30 seconds, and 72°C for 90 seconds), and a final elongation step at 95°C for 5 minutes. PCR products were verified and gel-purified as described earlier, and sequencing data obtained from Genewiz using the same primers that were used for PCR amplification. Sequences were then aligned against flagellin sequences whose antigenic type is known using the National Center for Biotechnology Information nucleotide collection database (https://blast.ncbi.nlm.nih.gov).

### Biofilm quantification.

We used the crystal violet assay to quantify the amount of biofilm synthesized by *Salmonella* strains.^15^ Briefly, overnight cultures were used to inoculate Hy-Soy lacking NaCl in quadruplicate wells of a 96-well plate. The plates were then incubated with shaking at 37°C for 48 hours. The wells were washed twice with PBS, and 100 μL of crystal violet staining solution (Sigma-Aldrich) was added into each well. The plates were incubated for 15 minutes at room temperature, before being washed five times with deionized water. The adherent material in the wells was destained by adding 200 μL of 70% ethanol into each well. Optical density at 600 nm was recorded using a plate reader (Molecular Devices, Sunnyvale, CA) and corrected by the optical density of the blank (medium only wells).

### Catalase assay.

We used the catalase assay developed by Iwase et al.^16^ with slight modifications. Bacterial strains were grown overnight in 1% soytone at 37°C with shaking. Optical density at 600 nm (OD_600_) was recorded to estimate bacterial concentration and 2 mL of culture (6–8 × 10^9^ bacteria) was centrifuged; the supernatant was removed; and the cell pellet was resuspended in 100 μL of normal saline (9 g/L sodium chloride; Quality Biological, Gaithersburg, MD) and transferred into 13 × 100 mm borosilicate glass tubes. One-hundred microliters of 1% Triton X-100 and 100 μL of 35% stabilized hydrogen peroxide were added. The tubes were left untouched at room temperature until the foaming stopped (about 2 minutes). The height of the foam in the tube was then recorded. A standard curve was also created using dilutions of commercially available bovine catalase (Sigma-Aldrich; 3,000 units/mg), and the final results were expressed as units of catalase (U) per 10^10^ bacterial cells, assuming an OD_600_ of 1.25 corresponds to 10^9^ bacteria/mL of culture for all strains tested.

## RESULTS

Of the 27 *Salmonella* strains that agglutinated with O:6 antisera and which were isolated from the blood of infants and children at l’Hôpital Gabriel Touré, Bamako, Mali, between 2002 and 2014, all were determined to be O:6,7 (serogroup C1) by agglutination with antisera and by MLST (Table 1). Sixteen isolates were identified as *S.* Paratyphi C (O:6,7;c;1,5), and all were Vi-positive. MLST revealed that although two strains belonged to ST146, the majority (14/16) belonged to the less common ST114.^12^ Five isolates were serovar Brazzaville (O:6,7;b;1,2) based on O- and H-agglutination. The H-type was confirmed by sequencing the *fliC* and *fljB* genes. We were not able to determine the ST as there was no entry for this combination of allele types in the *Salmonella* MLST database. The combination of alleles for all seven loci tested was identical for all five *S*. Brazzaville strains (Table 2), indicating that all five isolates of this serovar belonged to the same ST. Finally, four isolates were identified as *S.* Colindale (ST584) and two as *S.* Virchow (ST121 and ST755).

**Table 1:** Distribution of 27 *Salmonella* serogroup C1 strains isolated from the blood of febrile pediatric patients in Bamako, Mali, from 2002 to 2014

Serovar	O*	H*	ST†	No. of isolates
Paratyphi C	6,7	c;1,5	114	14
			146	2
Brazzaville	6,7	b;1,2	No assigned ST	5
Colindale	6,7	r;1,7	584	4
Virchow	6,7	r;1,2	121	1
			755	1

ST = sequence type.

* Determined by agglutination with antisera against O polysaccharide and H flagella antigens.

† Determined by amplifying and sequencing seven housekeeping genes according to Achtman et al.^12^

**Table 2:** Allele type of each locus from the five *S.* Brazzaville strains

	Locus						
Strain	*thrA*	*purE*	*sucA*	*hisD*	*aroC*	*hemD*	*dnaN*
J40	65	138	151	156	19	8	102
J58	65	138	151	156	19	8	102
J93	65	138	151	156	19	8	102
J94	65	138	151	156	19	8	102
J98	65	138	151	156	19	8	102

**Figure 1.**
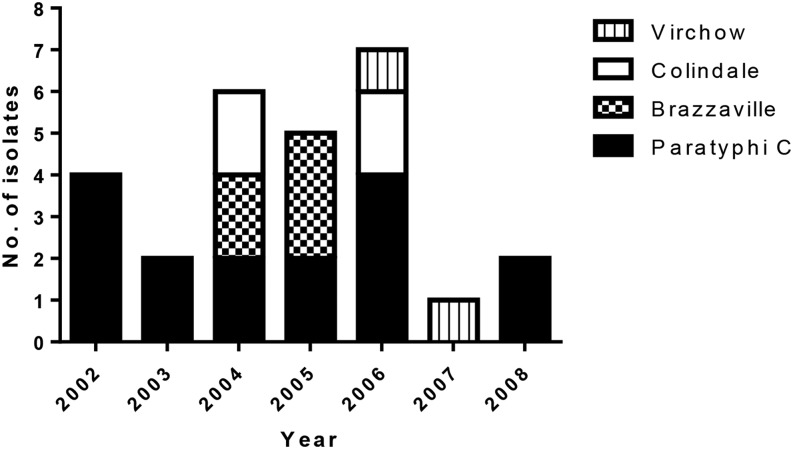
Number of isolates of each *Salmonella* serovar by year. Isolates were recovered from the blood of patients and assigned to a serovar by serum agglutination, multilocus sequence typing, and flagellin gene sequencing.

Of note, the five strains of *S*. Brazzaville were isolated within 10 months (April 2004 to February 2005; Figure 1), but were found in patients from four different communes (areas 2, 3, 5, and 6). In addition, *S.* Paratyphi C was isolated every year from 2002 to 2008 (except 2007).

**Figure 2.**
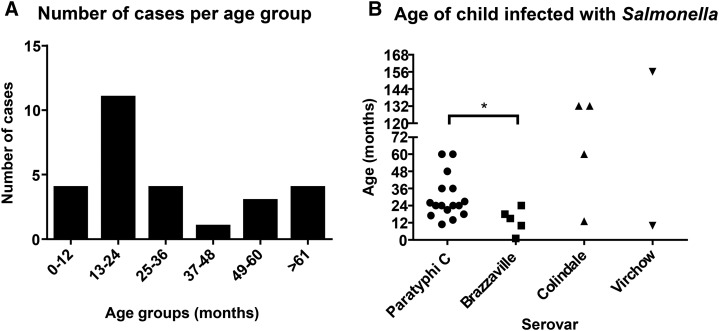
Distribution of *Salmonella* serogroup C1 nontyphoidal *Salmonella* cases by age (**A**) and serovar (**B**). *P = 0.03 (two-tailed *t* test).

Overall, children between 13 and 24 months of age had the most *Salmonella* serogroup C1 of all age groups (Figure 2A). *S.* Paratyphi C isolates were obtained from infants and children less than 6 years old, with a median age of 24 months (Figure 2B). *S.* Brazzaville strains were isolated from significantly younger children (median age of 15 months), with one strain isolated from a month-old newborn. *Salmonella* Colindale and *S.* Virchow infected children of a broader age range (10 months to 13 years). Clinical symptoms, diagnosis, and outcome for each patient are listed in Supplementary Table 1. Symptoms included fever, diarrhea, and chest indrawing, among others. The patients were mostly diagnosed with meningitis, septicemia, enteric fever, pneumonia, and malaria and there was one case of osteomyelitis. One patient, a 15-month-old child infected with *S.* Brazzaville and presenting with meningitis, died, whereas the rest of the patients recovered after hospitalization.

All 27 serogroup C1 isolates were sensitive to the 10 antibiotics we tested (amikacin, ampicillin, aztreonam, cefazolin, cefepime, streptomycin, ciprofloxacin, nalidixic acid, tigecycline, and sulfamethoxazole/trimethoprim).

**Figure 3.**
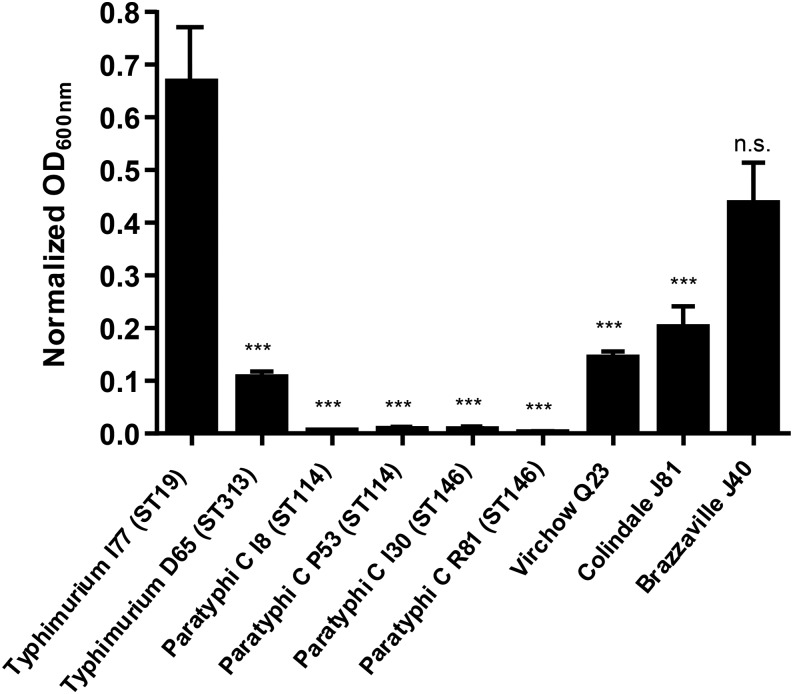
Biofilm formation in microtiter plates. The amount of biofilm formation was evaluated by the crystal violet binding assay and quantified by absorbance at 600 nm. Error bars indicate standard error of the mean, from at least four independent experiments. Asterisks indicate significant difference with *S.* Typhimurium I77. ***P < 0.001, n.s. = not significant (two-tailed *t* test).

The ability of *Salmonella* and other pathogenic bacteria to form biofilms has been linked to better persistence in the environment and increased resistance to environmental stresses and disinfection strategies.^17–21^ We compared biofilm-forming abilities of some of the *Salmonella* serogroup C1 strains isolated from Mali with two strains of *S.* Typhimurium also isolated from Mali: strain I77, which belongs to the most common ST of *S.* Typhimurium throughout the world, ST19; and strain D65, of ST313, which is circulating in sub-Saharan Africa.^22,23^ *Salmonella* Typhimurium I77 produced a strong biofilm, whereas strain D65 did not, as we have previously shown.^24^ None of the four tested *S.* Paratyphi C strains (two for each ST) produced biofilms under our experimental conditions (Figure 3). Strains of *S.* Virchow and *S.* Colindale produced less biofilms compared with *S*. Typhimurium I77. *Salmonella* Brazzaville produced more biofilms than the other tested serogroup C1 strains and was not statistically different from that of *S.* Typhimurium I77.

**Figure 4.**
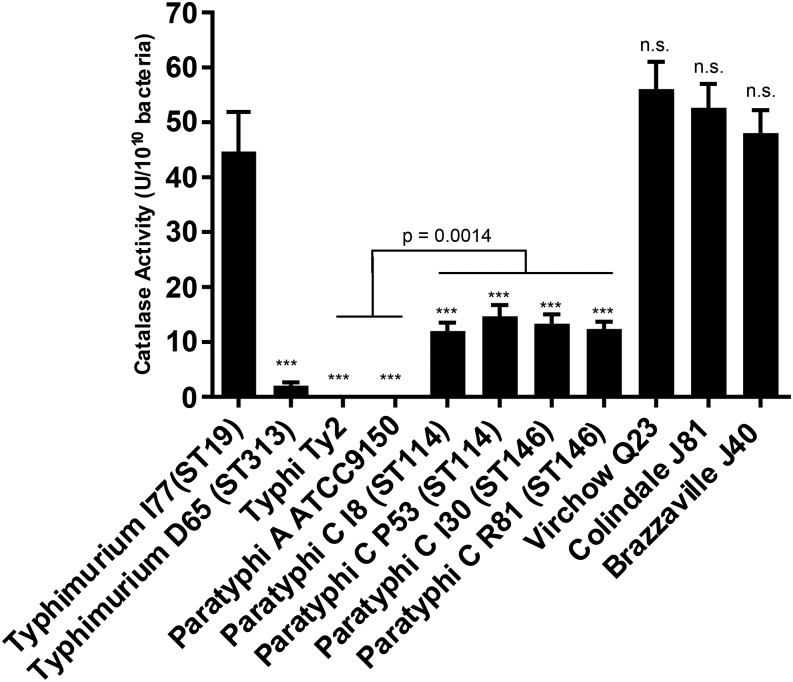
Catalase activity of various *Salmonella* strains. Catalase activity was evaluated for each strain in three independent experiments. Error bars indicate standard error of the mean. Asterisks indicate significant difference with *S.* Typhimurium I77. ***P < 0.001, n.s. = not significant (two-tailed *t* test).

Recently, reduced catalase activity has been reported for *S.* Typhimurium ST313, common in sub-Saharan Africa, compared with *S.* Typhimurium ST19, which is found worldwide.^25^ We analyzed the serogroup C1 strains isolated from Mali for their ability to degrade hydrogen peroxide and observed that all tested *S.* Paratyphi C strains had significantly lower catalase activity (between 12 and 15 U per 10^10^ bacteria) than *S.* Typhimurium I77 (ST19; 45 U per 10^10^ bacteria), regardless of the ST (ST114 or ST146) of the strains (Figure 4). That level was, however, higher in Paratyphi C than Typhi or Paratyphi A (*P* = 0.0014). By contrast, serovars Virchow, Colindale, and Brazzaville had very high catalase activity and were not statistically significantly different from *S.* Typhimurium ST19 (45 U per 10^10^ bacteria). Highly human host-adapted serovars Typhi and Paratyphi A had no detectable activity. As previously reported for ST313 strains, *S.* Typhimurium strain D65 had very low activity (2 U per 10^10^ bacteria).^26^

## DISCUSSION

The data presented here provide insight into the *Salmonella* serogroup C1 serovars that cause invasive disease in Mali. The predominance of *S.* Paratyphi C is particularly intriguing, as this serovar is rarely reported, either in African countries or in industrialized countries such as the United States where only 13 cases were reported between 1995 and 2012. Here, we described the identification of 16 isolates of this serovar from four different areas within Bamako, Mali, over a period of seven years (from 2002 to 2008), suggesting a widespread presence rather than a transient, localized outbreak. Historically, *S*. Paratyphi C has been associated with metastatic, suppurative infections. However, of the 16 *S*. Paratyphi C cases, only one had osteomyelitis. We hypothesize that the patients had atypical clinical presentations of *S*. Paratyphi C because of their immunocompromised status and other comorbidities. Children who are admitted to l’Hôpital Gabriel Touré can have malaria (highly prevalent), HIV (low prevalence), and/or are malnourished (very common). Six patients had malaria and one of these patients also had HIV. Interestingly, the most common ST was ST114, which differs from what Achtman et al. reported after analyzing 51 isolates of *S*. Paratyphi C from around the world: 34 were ST146 and only 3 were ST114.^12^ Similarly, the MLST database EnteroBase shows that the majority of *S*. Paratyphi C strains belong to ST146 (92 strains of 165 isolates deposited in the database).^27^ The situation in Mali is therefore unusual, both because of the number of cases in the surveillance period and in the distribution of the STs.

In addition, the presence of the very rare serovar *S*. Brazzaville, first identified 60 years ago in what is now the Democratic Republic of Congo, is surprising.^28,29^ It has historically been isolated from stool samples of patients with diarrhea as well as from pus of children with osteomyelitis.^30^ In the United States between 1995 and 2012, only 10 cases out of 698,763 NTS infections reported to the U.S. Centers for Disease Control and Prevention have been attributed to *S.* Brazzaville. Comorbidities could explain the higher-than-expected frequency of these otherwise rare serovars, as coinfections with HIV or malaria have been associated with a higher risk of contracting iNTS as well as higher mortality.^3,31,32^ Moreover, our previous study identified 21 isolates of the rare serovar *S.* Stanleyville (serogroup B) during the same surveillance period, suggesting that the particular environment in Mali allows for otherwise rare serovars to cause invasive disease in pediatric patients.^5^

Biofilm formation on gallstones has been linked to the persistence of *Salmonella* Typhi in the gallbladder of infected patients, leading to a chronic carrier stage.^33^ This has also been observed with *S.* Typhimurium in a murine gallbladder infection model.^34^ In a previous study, we observed that invasive *S.* Typhimurium ST313 strains isolated from the blood of Malian infants were poor biofilm producers.^24^ The amount of biofilm formed by ST313 strains was low and comparable to that of *S.* Typhi and *S.* Paratyphi A. This lack of the rdar (red, dry and rough; a hallmark of extracellular matrix components production) morphotype has also been documented for other serovars, such as Choleraesuis, and it was suggested to correlate with a more invasive disease phenotype.^35^ Our findings suggest that *S*. Paratyphi C is more similar to invasive *Salmonella* serovars than gastroenteritis-associated *Salmonella* in terms of biofilm production.

*Salmonella* Brazzaville showed no significant difference in biofilm production compared with *S.* Typhimurium I77 (ST19), suggesting that like I77, *S*. Brazzaville produces a biofilm and can survive desiccation.^24^ Moreover, these data suggest that this serovar may not be particularly invasive. None of the tested *S*. Paratyphi C strains were able to produce biofilms, which is consistent with previous observations that typhoidal (Typhi, Paratyphi A) and highly invasive nontyphoidal (Typhimurium ST313, Typhimurium var. Copenhagen, Choleraesuis) *Salmonella* are poor biofilm producers.^35^

Catalase activity has been linked to enhanced persistence in the environment, most likely by increasing survival during environmental stress. In addition, it has been suggested that catalase is required when bacteria are exposed to high concentrations of hydrogen peroxide, at high bacterial densities.^36,37^ Within *Salmonella*, loss of the ability to detoxify hydrogen peroxide is thought to be a marker of human host adaptation. Even though decreased catalase activity has been linked to increased susceptibility to oxidative stress, many articles report that human-adapted, invasive serovars tend to have lower or no catalase activity.^25^ Moreover, catalase-deficient mutants of *Salmonella* Typhimurium were shown to be as virulent as the wild-type strain in mice.^36^ We confirmed the low catalase activity of human host-adapted serovars, such as *S.* Typhi and *S.* Paratyphi, A. *S.* Colindale, *S.* Virchow, and *S.* Brazzaville displayed very high catalase activities that were not significantly different from *S*. Typhimurium I77, suggesting that they are poorly adapted to humans. *Salmonella* Paratyphi C showed significantly less catalase production than *S*. Typhimurium I77, but not as low as *S*. Typhi or *S*. Paratyphi A, suggesting that it may not be as human host-adapted as these typhoidal serovars.

In a previous study, we showed that most *S.* Typhimurium (serogroup B) and *S.* Enteritidis (serogroup D) strains isolated from Mali were highly resistant to several relevant antibiotics (ampicillin, chloramphenicol, and trimethoprim-sulfamethoxazole).^5^ Here, among 27 serogroup C1 strains isolated during the same surveillance period, none of these isolates were resistant to any of the 10 tested antimicrobials, suggesting that existing antibiotic treatments are currently sufficient to treat an NTS infection due to serogroup C1. However, several highly resistant serogroup C1 and C2 isolates, such as *S*. Newport and *S*. Kentucky, have been reported in other African countries, associated with both human and animal burden.^4,38^

This study emphasizes the need for broad surveillance of iNTS in Africa. Moreover, given the high diversity of serovars of human interest within serogroups C1 and C2 *Salmonella*, MLST can be used to help identify these serovars.^4^ In the case of the very rare *S.* Brazzaville, however, MLST was not useful as *S*. Brazzaville is absent from the *Salmonella* MLST database. Instead, a combination of methods including flagellin gene sequencing, biochemical testing, and traditional serum agglutination was required to identify this serovar.

## Supplementary Material

Supplemental Table.

## References

[1] Havelaar AH 2015 World Health Organization global estimates and regional comparisons of the burden of foodborne disease in 2010. PLoS Med 12: e1001923. 26633896 10.1371/journal.pmed.1001923 PMC4668832

[2] Feasey NA Dougan G Kingsley RA Heyderman RS Gordon MA, 2012 Invasive non-typhoidal *Salmonella* disease: an emerging and neglected tropical disease in Africa. Lancet 379: 2489–2499. 22587967 10.1016/S0140-6736(11)61752-2 PMC3402672

[3] Reddy EA Shaw AV Crump JA, 2010 Community-acquired bloodstream infections in Africa: a systematic review and meta-analysis. Lancet Infect Dis 10: 417–432. 20510282 10.1016/S1473-3099(10)70072-4 PMC3168734

[4] Fuche FJ Sow O Simon R Tennant SM, 2016 *Salmonella* Serogroup C: current status of vaccines and why they are needed. Clin Vaccine Immunol 23: 737–745. 27413069 10.1128/CVI.00243-16 PMC5014923

[5] Tapia MD 2015 Invasive nontyphoidal *Salmonella* infections among children in Mali, 2002–2014: microbiological and epidemiologic features guide vaccine development. Clin Infect Dis 61 (Suppl 4): S332–S338. 26449949 10.1093/cid/civ729 PMC4596934

[6] Tennant SM 2010 Identification by PCR of non-typhoidal *Salmonella enterica* serovars associated with invasive infections among febrile patients in Mali. PLoS Negl Trop Dis 4: e621. 20231882 10.1371/journal.pntd.0000621 PMC2834738

[7] Sow SO Diallo S Campbell JD Tapia MD Keita T Keita MM Murray P Kotloff KL Levine MM, 2005 Burden of invasive disease caused by *Haemophilus influenzae* type b in Bamako, Mali: impetus for routine infant immunization with conjugate vaccine. Pediatr Infect Dis J 24: 533–537. 15933564 10.1097/01.inf.0000164768.28135.0d

[8] Sow SO Tapia MD Diallo S Keita MM Sylla M Onwuchekwa U Pasetti MF Kotloff KL Levine MM, 2009 *Haemophilus influenzae* type b conjugate vaccine introduction in Mali: impact on disease burden and serologic correlate of protection. Am J Trop Med Hyg 80: 1033–1038. 19478272

[9] Campbell JD Kotloff KL Sow SO Tapia M Keita MM Keita T Diallo S Hormazabal JC Murray P Levine MM, 2004 Invasive pneumococcal infections among hospitalized children in Bamako, Mali. Pediatr Infect Dis J 23: 642–649. 15247603 10.1097/01.inf.0000130951.85974.79

[10] Felix A Pitt RM, 1951 The pathogenic and immunogenic activities of *Salmonella typhi* in relation to its antigenic constituents. J Hyg (Lond) 49: 92–110. 20475842 10.1017/s0022172400015394 PMC2234993

[11] Grimont PA Weill F-X, 2007. *Antigenic Formulae of the* Salmonella *Serovars* Paris, France: WHO Collaborating Center for Reference and Research on Salmonella, Institut Pasteur. Available at: http://www.scacm.org/free/Antigenic%20Formulae%20of%20the%20Salmonella%20Serovars%202007%209th%20edition.pdf. Accessed September 29, 2015.

[12] Achtman M 2012 Multilocus sequence typing as a replacement for serotyping in *Salmonella enterica*. PLoS Pathog 8: e1002776. 22737074 10.1371/journal.ppat.1002776 PMC3380943

[13] O’Farrell B Haase JK Velayudhan V Murphy RA Achtman M, 2012 Transforming microbial genotyping: a robotic pipeline for genotyping bacterial strains. PLoS One 7: e48022. 23144721 10.1371/journal.pone.0048022 PMC3483277

[14] Weill F-X Lailler R Praud K Kérouanton A Fabre L Brisabois A Grimont PAD Cloeckaert A, 2004 Emergence of extended-spectrum-β-lactamase (CTX-M-9)-producing multiresistant strains of *Salmonella enterica* serotype Virchow in poultry and humans in France. J Clin Microbiol 42: 5767–5773. 15583311 10.1128/JCM.42.12.5767-5773.2004 PMC535271

[15] Merritt JH Kadouri DE O’Toole GA, 2005. Growing and analyzing static biofilms. Curr Protoc Microbiol. 00: B:1B.1:1B.1.1–1B.1.17. 10.1002/9780471729259.mc01b01s00 PMC4568995 18770545

[16] Iwase T Tajima A Sugimoto S Okuda K Hironaka I Kamata Y Takada K Mizunoe Y, 2013 A simple assay for measuring catalase activity: a visual approach. Sci Rep 3: 3081. 24170119 10.1038/srep03081 PMC3812649

[17] Joseph B Otta SK Karunasagar I Karunasagar I, 2001 Biofilm formation by *Salmonella* spp. on food contact surfaces and their sensitivity to sanitizers. Int J Food Microbiol 64: 367–372. 11294359 10.1016/s0168-1605(00)00466-9

[18] Robijns SCA De Pauw B Loosen B Marchand A Chaltin P De Keersmaecker SCJ Vanderleyden J Steenackers HPL, 2012 Identification and characterization of 4-[4-(3-phenyl-2-propen-1-yl)-1-piperazinyl]-5H-pyrimido[5,4-b]indole derivatives as *Salmonella* biofilm inhibitors. FEMS Immunol Med Microbiol 65: 390–394. 22487085 10.1111/j.1574-695X.2012.00973.x

[19] Giaouris E 2015 Intra- and inter-species interactions within biofilms of important foodborne bacterial pathogens. Front Microbiol 6: 841. 26347727 10.3389/fmicb.2015.00841 PMC4542319

[20] Pande VV McWhorter AR Chousalkar KK, 2016 *Salmonella enterica* isolates from layer farm environments are able to form biofilm on eggshell surfaces. Biofouling 32: 699–710. 27268931 10.1080/08927014.2016.1191068

[21] Bridier A Sanchez-Vizuete P Guilbaud M Piard J-C Naïtali M Briandet R, 2015. Biofilm-associated persistence of food-borne pathogens. Food Microbiol 45: 167–178. 25500382 10.1016/j.fm.2014.04.015

[22] Okoro CK 2012 Intra-continental spread of human invasive *Salmonella* Typhimurium pathovariants in sub-Saharan Africa. Nat Genet 44: 1215–1221. 23023330 10.1038/ng.2423 PMC3491877

[23] Kingsley RA 2009 Epidemic multiple drug resistant *Salmonella* Typhimurium causing invasive disease in sub-Saharan Africa have a distinct genotype. Genome Res 19: 2279–2287. 19901036 10.1101/gr.091017.109 PMC2792184

[24] Ramachandran G Aheto K Shirtliff ME Tennant SM, 2016 Poor biofilm-forming ability and long-term survival of invasive *Salmonella* Typhimurium ST313. Pathog Dis 74: ftw049. 27222487 10.1093/femspd/ftw049 PMC5985484

[25] Singletary LA 2016 Loss of multicellular behavior in epidemic African nontyphoidal *Salmonella enterica* serovar Typhimurium ST313 strain D23580. MBio 7: e02265. 26933058 10.1128/mBio.02265-15 PMC4810497

[26] Yang J Barrila J Roland KL Kilbourne J Ott CM Forsyth RJ Nickerson CA, 2015 Characterization of the invasive, multidrug resistant non-typhoidal *Salmonella* strain D23580 in a murine model of infection. PLoS Negl Trop Dis 9: e0003839. 26091096 10.1371/journal.pntd.0003839 PMC4474555

[27] EnteroBase, 2017 EnteroBase. Available at: http://enterobase.warwick.ac.uk. Accessed January 13, 2017.

[28] Krubwa F Van Oye E Gatti F Vandepitte J, 1970 Epidemiologie de la Salmonellose a Kinshasa: role des porteurs sains et des aliments. Ann Soc Belg Med Trop 50: 319–338. 5514571

[29] Bernardo FMA Brandao CFSN, 1996 Enquete epidemiologique preliminaire sur les prevaevalences des *Salmonella* spp. a l’abattoir de Bissau (Guinee-Bissau). Rev Élev Méd Vét Pays Trop 49: 102–106. 9008957

[30] Vassiliadis P, 1960 *Salmonellae* of the Belgian Congo (8th report). Ann Soc Belg Med Trop 40: 423–428. 13780450

[31] Mahon BE Fields PI, 2016 Invasive Infections with nontyphoidal *Salmonella* in sub-Saharan Africa. Microbiol Spectr 4: 341–357. 10.1128/microbiolspec.EI10-0015-2016 27337467

[32] Falay D 2016 Microbiological, clinical and molecular findings of non-typhoidal *Salmonella* bloodstream infections associated with malaria, Oriental Province, Democratic Republic of the Congo. BMC Infect Dis 16: 271. 27286886 10.1186/s12879-016-1604-1 PMC4902913

[33] Crawford RW Rosales-Reyes R Ramírez-Aguilar M de la L Chapa-Azuela O Alpuche-Aranda C Gunn JS, 2010 Gallstones play a significant role in *Salmonella* spp. gallbladder colonization and carriage. Proc Natl Acad Sci USA 107: 4353–4358. 20176950 10.1073/pnas.1000862107 PMC2840110

[34] Adcox HE Vasicek EM Dwivedi V Hoang KV Turner J Gunn JS, 2016 *Salmonella* extracellular matrix components influence biofilm formation and gallbladder colonization. Infect Immun 84: 3243–3251. 27600501 10.1128/IAI.00532-16 PMC5067756

[35] Römling U Bokranz W Rabsch W Zogaj X Nimtz M Tschäpe H, 2003 Occurrence and regulation of the multicellular morphotype in *Salmonella* serovars important in human disease. Int J Med Microbiol 293: 273–285. 14503792 10.1078/1438-4221-00268

[36] Buchmeier NA Libby SJ Xu Y Loewen PC Switala J Guiney DG Fang FC, 1995 DNA repair is more important than catalase for *Salmonella* virulence in mice. J Clin Invest 95: 1047–1053. 7883952 10.1172/JCI117750 PMC441439

[37] Day WA Sajecki JL Pitts TM Joens LA, 2000 Role of catalase in *Campylobacter jejuni* intracellular survival. Infect Immun 68: 6337–6345. 11035743 10.1128/iai.68.11.6337-6345.2000 PMC97717

[38] Afema JA Byarugaba DK Shah DH Atukwase E Nambi M Sischo WM, 2016 Potential sources and transmission of *Salmonella* and antimicrobial resistance in Kampala, Uganda. PLoS One 11: e0152130. 26999788 10.1371/journal.pone.0152130 PMC4801205

