# Supplementary material for: Characterization of Invasive Salmonella Serogroup C1 Infections in Mali
Source: Am J Trop Med Hyg. 2017 Dec 26;98(2):589–94. doi: 10.4269/ajtmh.17-0508 (PMC5929196; doi:10.4269/ajtmh.17-0508)
Supplement: Supplementary file 1 [file tpmd170508.SD1.pdf]

Supplemental Table 1

Serotyping and clinical data associated with 27 serogroup C1 strains isolated from Mali

Strain	Date isolated	Name/code of the area of isolation	O-agglutination	H-agglutination	Metabolic test (when needed)	Sequence type (by MLST)	Serovar	Patient age (months)	Diagnosis	Outcome
P53	August 18, 2005	3	O:6,7	c;1,5	Dulcitol+, H_2S+	114	Paratyphi C	24	Pneumonia	Improved
P114	December 9, 2005	4	O:6,7	c;1,5	Dulcitol+, H_2S+	114	Paratyphi C	48	Malaria + HIV	Improved
P134	May 11, 2006	3	O:6,7	c;1,5	Dulcitol+, H_2S+	114	Paratyphi C	17	Enteric fever	Improved
P136	May 16, 2006	2	O:6,7	c;1,5	Dulcitol+, H_2S+	114	Paratyphi C	27	Septicemia	Improved
P159	August 7, 2006	1	O:6,7	c;1,5	Dulcitol+, H_2S+	114	Paratyphi C	11	Rhinobronchitis	Improved
I8	August 30, 2002	4	O:6,7	c;1,5	Dulcitol+, H_2S+	114	Paratyphi C	60	Pneumonia + Malaria	Improved
I30	October 16, 2002	Koulikoro	O:6,7	c;1,5	Dulcitol+, H_2S+	146	Paratyphi C	36	Pneumonia + Malaria	Improved
I40i	November 1, 2002	2	O:6,7	c;1,5	Dulcitol+, H_2S+	114	Paratyphi C	26	Pneumonia + Malaria	Improved
I46	November 8, 2002	4	O:6,7	c;1,5	Dulcitol+, H_2S+	114	Paratyphi C	36	Pneumonia	Improved
I75	February 3, 2003	6	O:6,7	c;1,5	Dulcitol+, H_2S+	114	Paratyphi C	24	Malaria	Unknown
I83	February 28, 2003	1	O:6,7	c;1,5	Dulcitol+, H_2S+	114	Paratyphi C	14	Meningitis	Unknown
J59	September 4, 2004	6	O:6,7	c;1,5	Dulcitol+, H_2S+	114	Paratyphi C	60	Enteric fever	Improved
J82	November 17, 2004	5	O:6,7	c;1,5	Dulcitol+, H_2S+	114	Paratyphi C	24	Osteomyelitis	Unknown
R77	April 12, 2008	4	O:6,7	c;1,5	Dulcitol+, H_2S+	114	Paratyphi C	24	Enteric fever	Improved
R81ii	April 19, 2008	1	O:6,7	c;1,5	Dulcitol+, H_2S+	146	Paratyphi C	21	Enteric fever	Improved
Q87	October 10, 2006	2	O:6,7	c;1,5	Dulcitol+, H_2S+	114	Paratyphi C	18	Enteric fever + Malaria + Bronchitis	Improved
J40	April 1, 2004	6	O:6,7	b;1,2*	nd	?	Brazzaville	15	Meningitis	Deceased
J58	September 1, 2004	5	O:6,7	b;1,2*	nd	?	Brazzaville	10	Pneumonia	Improved
J81	November 10, 2004	4	O:6,7	r;1,7	nd	584	Colindale	132	Diarrhea + Malaria	Improved
J84	November 27, 2004	6	O:6,7	r;1,7	nd	584	Colindale	132	Meningitis	Improved
J93	January 4, 2005	5	O:6,7	b;1,2*	nd	?	Brazzaville	18	Pneumonia	Unknown
J94	January 9, 2005	2	O:6,7	b;1,2*	nd	?	Brazzaville	1	Urinary tract infection	Improved
J98	February 7, 2005	3	O:6,7	b;1,2*	nd	?	Brazzaville	24	Malaria	Improved
Q23	October 19, 2006	6	O:6,7	r;1,2	nd	121	Virchow	156	Enteric fever + Malaria	Improved
Q30a	November 6, 2006	5	O:6,7	r;1,7	nd	584	Colindale	132	Malaria	Improved
Q31	November 8, 2006	4	O:6,7	r;1,7	nd	584	Colindale	60	Enteric fever + Malaria + Sickle-cell anemia	Improved
Q57	February 12, 2007	2	O:6,7	r;1,2	nd	755	Virchow	10	Septicemia + Hydronephrosis	Improved

MLST = multilocus sequence typing; nd = not determined.
* H-type was confirmed by PCR-amplifying and sequencing *fliC* and *fljB*.
